# Dynamics of Soil Organic Carbon and Aggregate Stability with Grazing Exclusion in the Inner Mongolian Grasslands

**DOI:** 10.1371/journal.pone.0146757

**Published:** 2016-01-11

**Authors:** Ding Wen, Nianpeng He, Jinjing Zhang

**Affiliations:** 1 Key Laboratory of Ecosystem Network Observation and Modeling, Institute of Geographic Sciences and Natural Resources Research, Chinese Academy of Sciences, Beijing 100101, China; 2 University of Chinese Academy of Sciences, Beijing 100049, China; 3 College of Resource and Environmental Science, Jilin Agricultural University, Changchun 130118, China; Banaras Hindu University, INDIA

## Abstract

Grazing exclusion (GE) has been deemed as an important approach to enhance the soil carbon storage of semiarid grasslands in China; however, it remains unclear how different organic carbon (OC) components in soils vary with the duration of GE. Here, we observed the changing trends of different OC components in soils with increased GE duration in five grassland succession series plots, ranging from free grazing to 31-year GE. Specifically, we measured microbial biomass carbon (MBC), easily oxidizable OC (EOC), water-soluble OC (WSOC), and OC in water stable aggregates (macroaggregates [250–2000 μm], microaggregates [53–250 μm], and mineral fraction [< 53 μm]) at 0–20 cm soil depths. The results showed that GE significantly enhanced EOC and WSOC contents in soils, but caused a decline of MBC at the three decade scale. Macroaggregate content (F = 425.8, *P* < 0.001), OC stored in macroaggregates (F = 84.1, *P* < 0.001), and the mean weight diameter (MWD) of soil aggregates (F = 371.3, *P* < 0.001) increased linearly with increasing GE duration. These findings indicate that OC stored in soil increases under three-decade GE with soil organic matter (SOM) stability improving to some extent. Long-term GE practices enhance the formation of soil aggregates through higher SOM input and an exclusion of animal trampling. Therefore, the practice of GE may be further encouraged to realize the soil carbon sequestration potential of semi-arid grasslands, China.

## Introduction

Some management practices, such as the promotion of native vegetation growth, sowing of legumes and grasses, and decreasing grazing intensity may enhance soil organic carbon (SOC) storage in grasslands [1–3]. Grazing exclusion (GE) represents as an important approach to improve grasslands in Inner Mongolia, China, and has been widely implemented since 2000. Previous studies have demonstrated that typical grasslands subjected to GE have an enormous capacity to enhance SOC storage at decadal scale [3–8], and the stability of newly formed SOC under GE has been explored using soil aggregates [9] and soil particle-size fractions [10].

Physical fractionation is widely used to study the storage and turnover of soil organic matter (SOM), because it incorporates three levels of analysis: SOM structural and functional complexity, and the linkage to functioning [11–15]. Soil aggregates, which are the secondary organomineral complexes of soil, are important for the physical protection of SOM. Thus, changes in soil aggregates may be used to characterize the impacts of management strategies on soil quality, including soil porosity, aeration, water retention, and erodibility [13]. Organic carbon (OC) stored in macroaggregates has a stronger response to land-use change than that of SOC, and may be used as an important diagnostic indicator for the potential changes [16,17]. To some extent, the protection of macroaggregates is considered to be fundamental for sustaining high SOC storage, and has been used in many ecological models [9,11,16–19].

Changes in different OC components of soils provide information in advance about how SOC storage changes with land-use change. Sequeira and Alley [20] investigated the possibility of particulate organic matter (POM), free light fraction (FLF), and easily oxidizable carbon (EOC) as sensitive indices to reflect how changes in management affect soil characteristics, and found that FLF was the most sensitive parameter. Leifeld and Kogel-Knabner [21] found that OC stored in water stable aggregates (>20 μm) is an appropriate early indicator of land-use changes. Scientists explored how OC content varies in relation to different soil fractions under different management practices [9,22,23]. Some recent studies have investigated how decadal GE influences OC in soil aggregates and other fractions (e.g., FLF), with a strong focus on aggregate stability and physical protection the in Inner Mongolian grasslands [9,24]. However, it remains unclear how OC stored in soil aggregates of different sizes and stability alter with increasing GE duration.

In this study, we measured the contents of SOC, microbial biomass carbon (MBC), EOC, water-soluble organic carbon (WSOC), and OC in soil aggregates using five plots with different GE histories (from free grazing to 31-yr GE). The main goals of this study were to (1) assess the influences of GE duration on different OC components stored in soil; (2) explore the changing trends of different OC components stored in soil with increasing GE duration.

## Materials and Methods

### Study site

**Fig 1:**
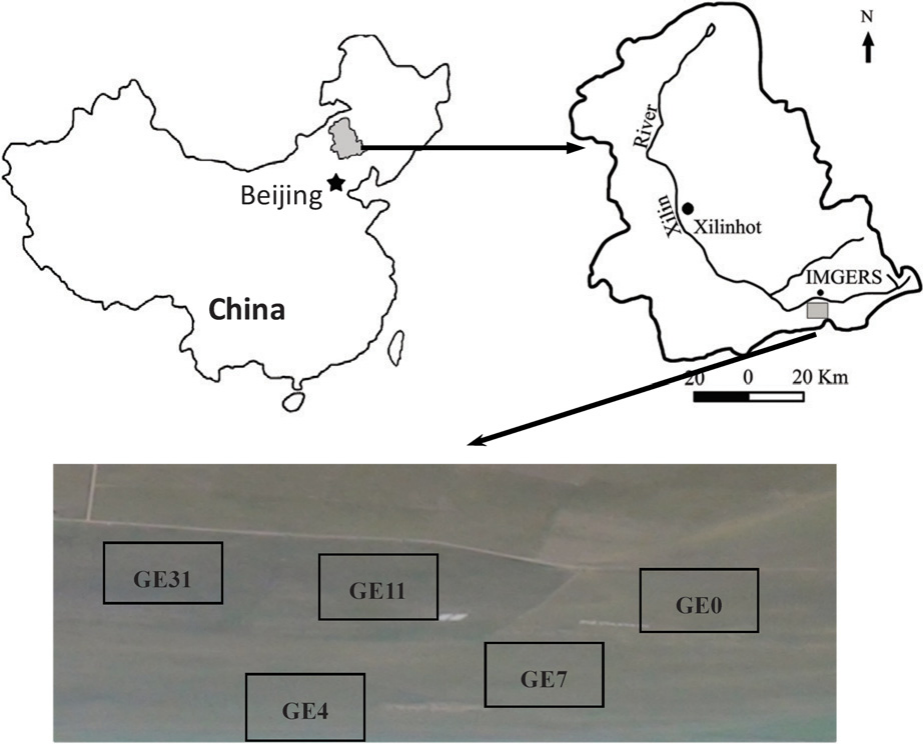
Experimental area and the five experimental plots. **IMGERS, Inner Mongolia Grassland Ecosystem Research Station (43°33′N, 116°40′E).** GE0, free grazing grassland; GE4, 4-yr grazing exclusion grassland; GE7, 7-yr grazing exclusion grassland; GE11, 11-yr grazing exclusion grassland; GE31, 31-yr grazing exclusion.

Field sampling was carried out on a typical steppe on the Mongolian Plateau (43°33′N, 116°40′E; Fig 1), which is administered by Mongolia Grassland Ecosystem Research Station (IMGERS), Chinese Academy of Sciences, China. The area belongs to a typical semi-arid continental climate with a mean annual temperature of 1.1°C and an average annual precipitation of 345 mm from 1980 to 2010 [6]. The soil is of the chestnut type, or Calcic Chernozems [25], which developed from Aeolian sediments. The soils are characterized by rich sand content, in the range of 60% to 75% [10]. Soil pH varies from 7.2 to 8.2 in the surface soils. Based on 30 year monitoring data, the vegetation of the region is dominated by grassland plant species, such as *Leymus chinensis* (44.5%, relative biomass), *Stipa grandis* (34.0%), and *Cleistogenes squarrosa* (8.7%) [6].

### Experimental design

The succession chronosequence consisted of five selected grasslands that were selected based on their different GE histories, named GE0, GE4, GE7, GE11, and GE31 (Table 1 and Fig 1). Plot GE0 has been subject to long-term free-grazing by sheep and is in good condition based on the aboveground plant community and diversity [26]. Plot GE4 was established in 2008 by fencing off a section of previously free-grazed grassland. Plots GE7, GE11, and GE31 were similarly fenced in 2004, 1999, and 1979, respectively. These GE plots ranged from 0.8 (GE4) ha to 24 ha (GE31) in area, and were floristically and topographically similar, and were distributed across a 2 km × 2 km area [27] (Fig 1). Historically, these plots had not fertilized at any way. The aboveground biomass significantly differed across GE0 and long-term GE plots (ranging from 51.2 to 148.4 g m^–2^). Therefore, changes in the soil properties in these plots (Table 1) were primarily due to the influence of grazing intensity and GE duration on new organic matter input by plants and SOM turnover (Table 1).

**Table 1:** Changes in soil properties with grazing-exclusion duration in in the Inner Mongolian grasslands.

	Soil organic carbon (SOC, g kg^–1^)	Easily oxidizable carbon (EOC, g kg^–1^)	Water-soluble organic carbon (WSOC, g kg^–1^)	Microbial biomass carbon (MBC, mg kg^–1^)	Total nitrogen (TN, g kg^–1^)	Total phosphorus (TP, g kg^–1^)	pH	Land-use history
Free grazing grassland (GE0)	14.36 ± 1.26 ^a^^†^	2.84 ± 0.19 ^a^	0.27 ± 0.02 ^a^	47.52 ± 0.46^a^	1.43 ± 0.10	0.224 ± 0.016 ^a^	8.17 ± 0.29 ^a^	Long-term free-grazing, good condition ^‡^
4-yr grazing exclusion (GE4)	14.31 ± 0.61 ^a^	7.62 ± 0.34 ^b^	0.29 ± 0.02 ^ab^	42.69 ± 1.73 ^ab^	1.60 ± 0.03	0.271 ± 0.004 ^b^	8.07 ± 0.11 ^a^	Grassland fenced since 2008, good condition
7-yr grazing exclusion (GE7)	15.03 ± 0.96 ^a^	7.55 ± 0.17 ^b^	0.30 ± 0.01 ^b^	38.06 ± 1.59 ^b^	1.64 ± 0.16	0.300 ± 0.011 ^b^	7.92 ± 0.16 ^ab^	Grassland fenced since 2004, good condition
11-yr grazing exclusion (GE11)	17.23 ± 1.27 ^b^	9.38 ± 0.29 ^c^	0.32 ± 0.02 ^bc^	39.53 ± 1.89 ^b^	1.72 ± 0.10	0.298 ± 0.002 ^c^	7.66 ± 0.19 ^b^	Grassland fenced since 1999, good condition
31-yr grazing exclusion (GE31)	19.95 ± 0.27 ^c^	12.8 ± 1.12 ^d^	0.34 ± 0.01 ^c^	40.02 ± 0.66 ^b^	1.48 ± 0.72	0.284 ± 0.009 ^c^	7.19 ± 0.29 ^c^	Grassland fenced since 1979, good condition
F-value	18.73 ^**^	125.61^**^	6.82 ^**^	5.09 ^*^	0.86 ^NS^	50.70 ^**^	15.57 ^**^	

^†^ Data are represented as mean ± 1 SD, and data with the same letters within each column indicate no significant difference at *P* = 0.05 level.

* *P* <0.05

** *P* <0.01

NS no significance at *P* = 0.05.

^‡^ All plots were not subjected to any fertilization treatments

### Soil sampling and analysis

Field sampling was conducted at the end of July 2011. In each plot, three sampling quadrats (1 × 1 m) were established at random. Aboveground biomass was clipped at ground level, and litter was subsequently collected. Soil samples in the 0–20 cm soil layer were collected from 10 points in each quadrat using soil sampler (diameter, 8 cm). Thus, three soil samples (with each sample weighting >2.5 kg) were collected from each plot. In the laboratory, we first removed the roots and apparent organic debris from the soil samples by hand. Then, approximately 100 g of each soil samples was air-dried in a ventilation room to analyze the soil properties, while the remainder was stored at 4°C. Soil bulk density was measured using soil cores (volume, 100 cm^3^) at depths of 0–20 cm, with 3 replicates for each plot within these sampling quadrats.

The OC content (%) was measured in all samples using a modified Mebius method [28]. The content of MBC in fresh soil samples was analyzed using the fumigation-extraction method [29], in which OC content in 0.5 M K_2_SO_4_ soil extract was analyzed using a TOC Analyzer (Elementar, Germany). EOC in soils was measured using the method of Blair et al. [30]. In brief, 15 mg air-dried soil was incubated with 333 mmol l^–1^ KMnO_4_ solution for 1 hour, and the amount of EOC was determined spectrophotometrically from the amount of KMnO_4_ that had been reduced (565 nm). WSOC was extracted from 10 g of fresh soil with a soil to water ratio of 1:2 at 25°C. After shaking for 0.5 h at 250 r min^–l^, and then centrifuging for 10 minutes at 15 000 r min^–l^, the supernatant was filtered using a 45 μm carbon-free membrane. The filtrate was measured with a TOC analyzer. We did not measure soil inorganic C (SIC) content in samples, because our previous study have demonstrated that SIC is relatively lower and ranges from 0 to 0.38 g kg^–1^ in the 0–20 cm soil layer in plot GE11, even though SIC may reach 5.0 g kg^–1^ in the 50–100 cm soil layer [31].

Soil aggregates were separated into different sizes following the method of six *et al*.[11]. In brief, three 150-g subsamples of fresh soil (<2 mm diameter) were used to analyze the size distribution of aggregates, and a sieve system with 250 μm and 53 μm sieves was moved up and down in distilled water at a rate of 50 strokes in 2 min. Aggregates were physically separated into three aggregate sizes: (1) macroaggregates of 250 to 2000 μm in diameter, (2) microaggregates of 53 to 250 μm in diameter, and (3) mineral soil of <53 μm in diameter. After wet sieving, all of the samples were oven-dried at 65°C. OC content was measured for each aggregate size class using a modified Mebius method [28].

C storage (g C m^−2^) in soil and soil aggregates was calculated as follows:

$$C_{\text{storage}}=C_{\text{content}}\times M_{\text{i}}\times B\times D\times S÷10$$ (1)

where *C*_content_ represents OC content in the soil or soil aggregates (g kg^−1^), *M*_i_ is the content of each soil aggregate (g kg^−1^), B is bulk density (g cm^−3^), *D* is layer thickness (cm), and *S* is the cross-sectional area (m^2^).

Aggregate stability of soils was expressed as the mean weight diameter of aggregates (MWD) as described by Kuo [32]:

$$MWD=\sum_{i=1}^{n}X_{i}\times \frac{WSA_{i}}{100}$$ (2)

where *X* is the mean diameter of each aggregate (*i*), *WSA*_i_ is the proportion of the total sample weight recovered of a given aggregate size after wet sieving (*i*), and n is the number of aggregate sizes.

Soil total nitrogen content (TN, %) was measured using the modified Kjeldahl wet digestion procedure [33], using a 2300 Kjeltec Analyzer Unit (FOSS, Sweden). Total phosphorus (TP) concentrations was determined by the ammonium molybdate method after persulfate oxidation [34]. Soil pH was determined using a pH meter in soil mixed with distilled water at a ratio 1:2.5.

### Statistical analysis

The measured data were provided in the supplementary file (S1 Dataset). One-way analysis of variance (One-ANOVA) with Duncan’s post-hoc test for multiple comparisons was used to determine how GE influences the different OC components stored in soil. Pearson correlations (2-tailed test) were used to explore the correlation among different OC components stored in soil and other soil properties (TN, TP, and pH). Curve regression was used to identify how OC content was related to various soil components (e.g., SOC, SWOC, EOC, MWD, and others) and GE duration. Data are represented as the mean ± 1 standard deviation (n = 4). Differences were considered to be significant at the *P* = 0.05 level. All analyses were conducted using SPSS statistical software (v. 13.0, SPSS, Chicago, IL, USA).

## Results

**Fig 2:**
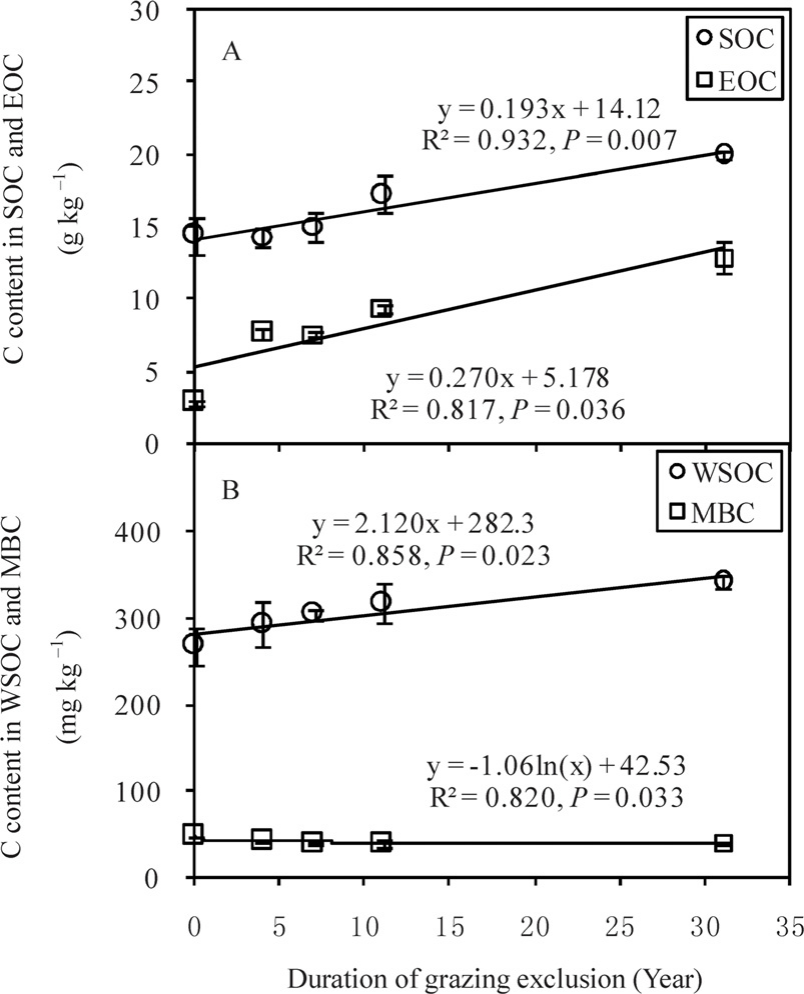
Changes in soil organic carbon (SOC), easily oxidizable organic carbon (EOC) (A), water-soluble organic carbon (WSOC), and microbial biomass carbon (MBC) (B) with grazing-exclusion duration in the Inner Mongolian grasslands.

The results showed that SOC, EOC, WSOC, and MBC significantly differed among the five plots (F = 18.73, *P* < 0.01 for SOC; F = 5.09, *P* < 0.05 for MBC; F = 125.61, *P* < 0.01 for EOC; F = 6.82, *P* < 0.01 for WSOC) (Table 1). SOC, EOC, and WSOC linearly increased with increasing GE duration (Fig 2). In contrast, MBC decreased with increasing GE duration. Furthermore, significantly positive correlations were observed among SOC, EOC, and WSOC (Table 2). However, MBC was significantly negatively correlated with EOC and WSOC.

**Table 2:** Pearson correlation coefficients among organic carbon components and other soil properties.

	SOC ^†^	EOC	WSOC	MBC	TN	TP	pH
SOC	1	0.811^**^ ^‡^	0.663^**^	-0.260 ^NS^	0.311 ^NS^	0.693^**^	-0.243 ^NS^
EOC		1	0.851^**^	-0.612^**^	0.403 ^NS^	0.709^**^	-0.096 ^NS^
WSOC			1	-0.592^*^	0.252 ^NS^	0.497 ^NS^	0.082 ^NS^
MBC				1	-0.598 ^*^	-0.584^*^	0.020 ^NS^
TN					1	0.428^*^	0.119 ^NS^
TP						1	-0.477 ^*^
pH							1

^†^ see Table 1 for abbreviations.

^‡^ * *P* < 0.05

** *P* < 0.01

NS no significance at *P* = 0.05.

**Fig 3:**
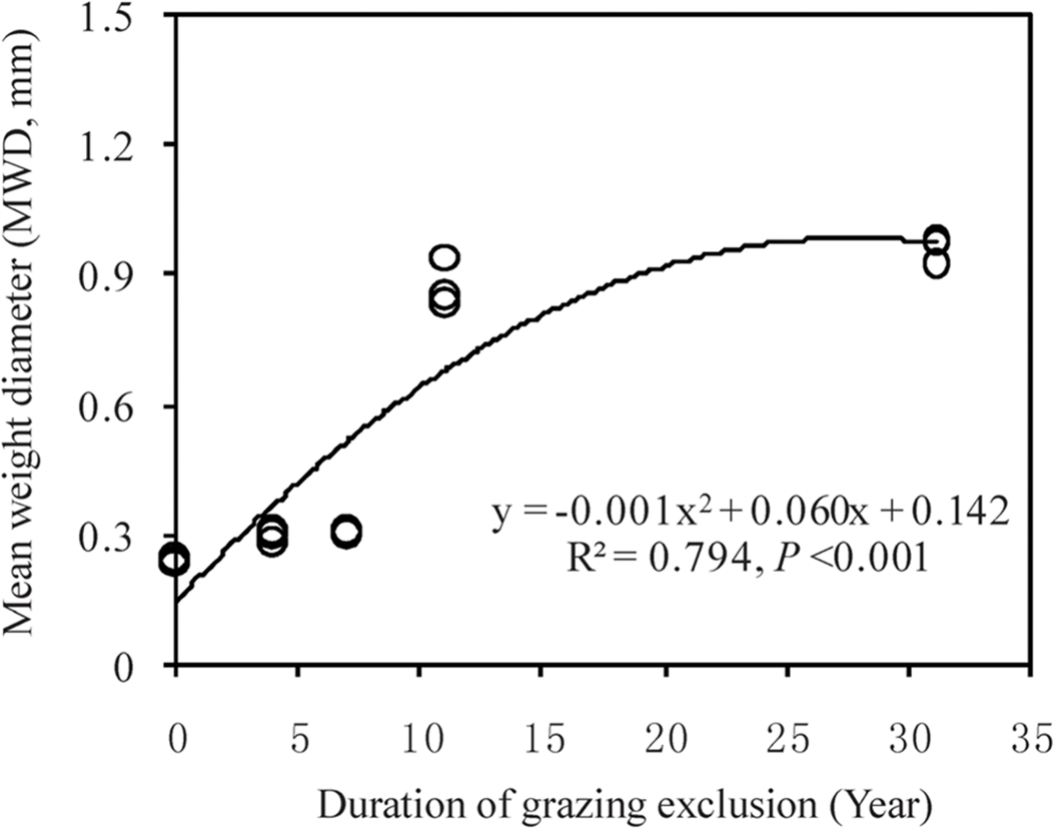
Changes in mean weight diameter with grazing-exclusion duration in the Inner Mongolian grasslands.

The relative proportion of various aggregate size classes is influenced by GE. In detail, proportion of macroaggregates significantly increased after GE was implemented (R^2^ = 425.79, *P* < 0.01), ranging from 14.8% in GE0 to 82.2% in GE31 (Table 3). Proportion of microaggregates (R^2^ = 84.77, *P* < 0.01) and mineral soils (R^2^ = 129.5, *P* < 0.01) decreased significantly with increased GE duration. The aggregate stability of soils, expressed as MWD, was significantly different among different plots (F = 371.32, *P* < 0.001), ranging from 0.24 in GE0 to 0.96 in GE31, showing a binomial increase with GE duration (R^2^ = 0.794, *P* < 0.001) (Fig 3).

**Table 3:** Changes in the content of soil aggregates and their organic carbon content with grazing-exclusion duration in the Inner Mongolian grasslands.

	Soil aggregation (%)			Carbon content in soil aggregation (gC kg^–1^)			Aggregate stability
	Macroaggregate	Microaggregate	Mineral fraction	Macroaggregate	Microaggregate	Mineral fraction	Mean weight diameter (MWD)
Free grazing grassland (GE0)	14.8 ± 0.50 ^a^^†^	52.5 ± 0.84 ^a^	32.3 ± 0.46 ^a^	2.62 ± 0.28 ^a^	5.59 ± 0.52 ^a^	6.07 ± 0.48 ^a^	0.24 ± 0.01 ^a^
4-yr grazing exclusion (GE4)	19.3 ± 1.52 ^ab^	53.5 ± 5.01 ^a^	26.3 ± 4.74 ^b^	2.98 ± 0.08 ^a^	6.88 ± 0.61 ^b^	4.97 ±1.12 ^b^	0.29 ± 0.01 ^ab^
7-yr grazing exclusion (GE7)	21.2 ± 0.64 ^b^	42.5 ± 1.19 ^b^	35.7 ± 1.46 ^c^	3.26 ± 1.35 ^a^	5.72 ± 0.91 ^ab^	8.51 ±0.48 ^c^	0.30 ± 0.01 ^b^
11-yr grazing exclusion (GE11)	74.7 ± 5.34 ^c^	16.5 ± 5.42 ^c^	7.63 ± 0.99 ^d^	15.5 ± 2.50 ^b^	2.42 ± 0.61 ^c^	1.62 ±0.10 ^d^	0.87 ± 0.06 ^c^
31-yr grazing exclusion (GE31)	82.2 ± 2.64 ^d^	14.6 ± 2.84 ^c^	1.88 ± 0.59 ^e^	16.1 ± 0.82 ^b^	2.47 ± 0.48 ^c^	0.42 ±0.14 ^e^	0.96 ± 0.03 ^d^

^†^ Data are represented as mean ± 1 SD, and data with the same letters within each column indicate no significant difference at *P* = 0.05 level.

**Fig 4:**
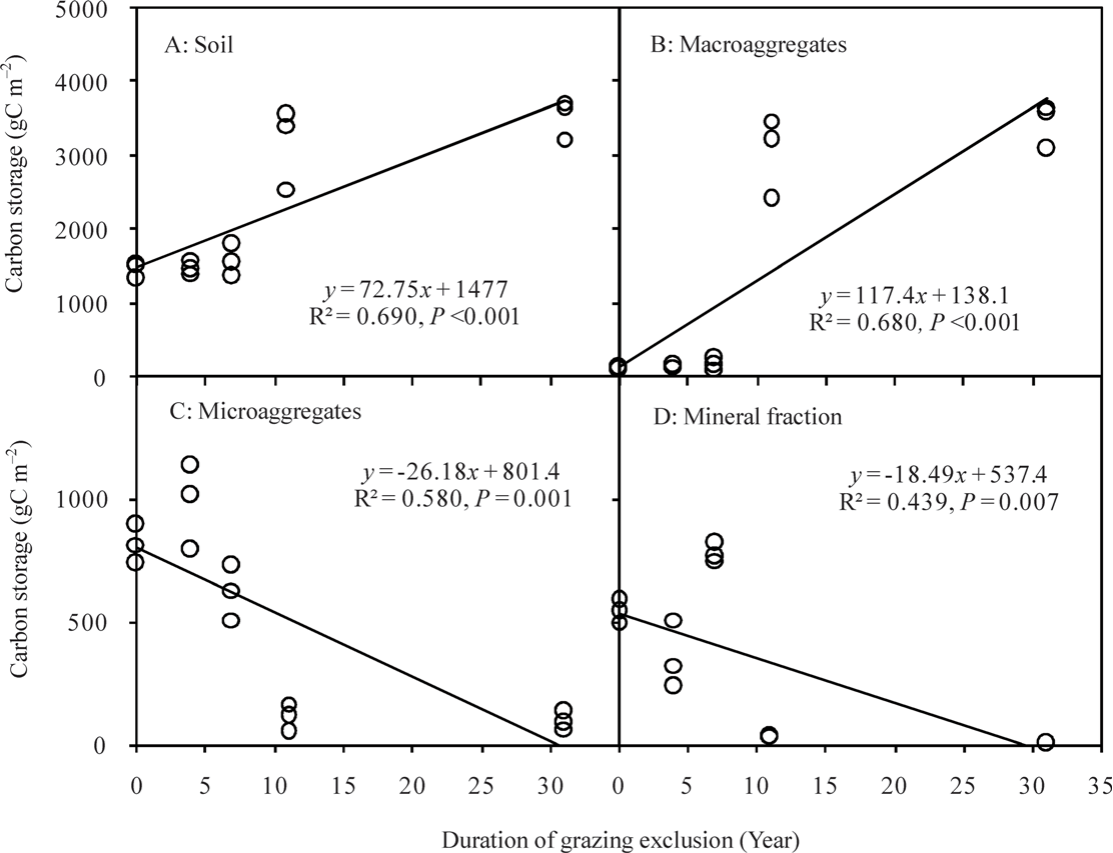
Carbon storage in soil aggregates with grazing-exclusion duration in the Inner Mongolian grasslands.

SOC storage in the 0–20 cm soil layer varied significantly, ranging from 1462.9 g C m^–2^ in GE0 to 3542.5 g C m^–2^ in GE31 (F = 36.2, *P* < 0.01). C storage in macroaggregates significantly increased from 107.7 g C m^–2^ in GE0 to 3444.6 g C m^–2^ in GE31 (F = 105.4, *P* < 0.01) (Table 3). However, C storage in microaggregates (F = 46.4, *P* < 0.01) and mineral soils (F = 72.2, *P* < 0.01) significantly decreased with increasing GE duration. Regression analysis showed that C storage both in the soil and in the macroaggregates increased linearly with increasing GE duration (Fig 4). In contrast, C storage in microaggregates and mineral soils linearly decreased with increasing GE duration.

## Discussion

The practice of GE enhanced SOC storage at the decadal scale in the Inner Mongolian grasslands. The increases of SOC storage were mainly observed in macroaggregates while SOC storage in microaggregates decreased to some extent. These findings were consistent with previous studies [6,35]. For instance, a meta-analysis of 133 published papers by Wang *et al*. [3] showed that the practices of GE increase SOC content by 34% on average in the Inner Mongolian grasslands. He *et al*. [7] compared the influence of different grassland managements on SOC storage in this region and found that SOC storage was enhanced in the order of GE > mowing > winter grazing > reclamation. Previously we examined the effects of different grazing intensities in the Inner Mongolia grasslands (0, 1.5, 3.0, 4.5, 6.0, 7.5, and 9.0 sheep ha^–1^), and found that SOC storage enhanced significantly in grasslands with no grazing or low grazing pressure but decreased under heavy grazing pressure [36]. Overall, GE enhanced SOC storage in the surface soils of the Inner Mongolian grasslands at decadal scales through increasing new SOM input and depressing SOM decomposition to some extent [3–5,27,37].

Decadal GE significantly enhanced EOC and WSOC content in the surface soils, with a clear linear relationship. Some studies have demonstrated that EOC and WSOC are sensitive to management or restoration practices, and may be used as indicators for the changes in soil quality [18,38]. Embacher *et al*. [39] reported that EOC in the 0–30 cm soil layer ranges between 34 and 42 mg kg^–1^ with noticeable seasonality. Jiang and Xu [40] reported that the quality and seasonality of litter input generally affected the seasonal dynamics of EOC, MBC, and WSOC. Therefore, seasonal investigations are required to improve our understanding about how decadal GE influences soil quality, however, one-time sampling at the peak period of aboveground biomass is a reliable indicators of the general trends for the alteration of SOM components among different plots.

MBC decreased linearly with increasing GE duration. Similar results were reported by Wu *et al*. [41], who found that decadal GE grasslands had lower MBC and soil respiration rates than grazing grasslands in Inner Mongolia. The decrease in soil temperature in decade GE grasslands due to increasing accumulation of aboveground litter [26], is one plausible explanation for the observed decrease in MBC. Furthermore, the decrease in MBC or changes in soil microbial communities with the succession of GE grasslands may lead to a decrease in soil respiration rates [27]. Therefore, the depression of soil respiration rates by decreasing MBC and soil temperature may be another important mechanism for enhancing SOC storage in three-decade GE grasslands [6,8].

Land-use change strongly influences various soil properties such as SOC storage and aggregate structure [12,20]. Our findings showed that both OC content and storage in macroaggregates increased linearly after initiating GE (Fig 4 and Table 3). Compared with continuously grazed grasslands, the higher macroaggregates in three-decade GE grasslands resulted from the greater input of organic matter acting as binding agents in combination with an exclusion of animal trampling [9,24]. Thus, GE practices promoted the physical protection of SOM by increasing soil macroaggregation, representing a viable management option to enhance SOC storage in Inner Mongolian grasslands. Su *et al*. [42] demonstrated that conversion from annual crop to alfalfa increased significantly OC stored in macroaggregates in sandy grasslands of Inner Mongolia. However, Six *et al*. [43] and Lisboa *et al*. [18] found that soil microaggregates were more sensitive to land-use changes. Furthermore, the shift from forest to grassland has no noticeable effect on macroaggregates due to a percentage shift in the distribution of OC among different soil aggregates, and such shifts may delay or decrease the loss of OC caused by land-use changes [44,45].

As expected, soil macroaggregate content, OC storage, and MWD increased with GE duration, indicating that SOM stability increases under three-decade GE treatment due to the greater physical protection of SOM by soil aggregates [11,13,46]. Similarly, Steffens *et al*. [24] and Wiesmeier *et al*. [9] reported that two-decades of GE improved soil macroaggregate structure, whereas heavy grazing deteriorated soil macroaggregate structure in the Inner Mongolian grasslands. As proposed by previous studies for cropland ecosystems [16,47,48], the majority of SOC accumulated from new C inputs is preferentially sequestered in microaggregates and macroaggregates. Hence, changes in macroaggregate and microaggregates serve as ideal indicators of soil C sequestration potential in terrestrial ecosystems. The practice of three-decade GE promoted the physical protection of SOM by increasing soil aggregation, representing a management option to enhance soil C sequestration potential in Inner Mongolia grasslands.

## Conclusions

Three-decade GE significantly enhanced SOC, EOC and WSOC content in the 0–20 cm soil layer in the grasslands of Inner Mongolia. Furthermore, long-term GE significantly enhanced soil macroaggregate content, OC storage, and MWD with increasing GE duration. These findings indicated that three-decade GE grasslands improved SOM stability through increasing macroaggregates. The practice of GE therefore should be encourage to restore the vegetation of degraded grasslands in Inner Mongolia and to enhance SOC storage and its stability.

## Supporting Information

S1 Dataset (XLS)
